# Supplementary material for: Cortical gene expression architecture links healthy neurodevelopment to the imaging, transcriptomics and genetics of autism and schizophrenia
Source: Nat Neurosci. 2024 Apr 22;27(6):1075–86. doi: 10.1038/s41593-024-01624-4 (PMC11156586; doi:10.1038/s41593-024-01624-4)
Supplement: Supplementary file 2 — Reporting Summary [file 41593_2024_1624_MOESM2_ESM.pdf]

nature portfolio

Corresponding author(s): Richard Dear

Last updated by author(s): February 9, 2024

Reporting Summary

Nature Portfolio wishes to improve the reproducibility of the work that we publish. This form provides structure for consistency and transparency in reporting. For further information on Nature Portfolio policies, see our Editorial Policies and the Editorial Policy Checklist.

Statistics

For all statistical analyses, confirm that the following items are present in the figure legend, table legend, main text, or Methods section.

n/a	Confirmed	
☐	☒	The exact sample size (*n*) for each experimental group/condition, given as a discrete number and unit of measurement
☐	☒	A statement on whether measurements were taken from distinct samples or whether the same sample was measured repeatedly
☐	☒	The statistical test(s) used AND whether they are one- or two-sided<br>*Only common tests should be described solely by name; describe more complex techniques in the Methods section.*
☒	☐	A description of all covariates tested
☐	☒	A description of any assumptions or corrections, such as tests of normality and adjustment for multiple comparisons
☐	☒	A full description of the statistical parameters including central tendency (e.g. means) or other basic estimates (e.g. regression coefficient) AND variation (e.g. standard deviation) or associated estimates of uncertainty (e.g. confidence intervals)
☐	☒	For null hypothesis testing, the test statistic (e.g. *F*, *t*, *r*) with confidence intervals, effect sizes, degrees of freedom and *P* value noted<br>*Give P values as exact values whenever suitable.*
☒	☐	For Bayesian analysis, information on the choice of priors and Markov chain Monte Carlo settings
☒	☐	For hierarchical and complex designs, identification of the appropriate level for tests and full reporting of outcomes
☐	☒	Estimates of effect sizes (e.g. Cohen's *d*, Pearson's *r*), indicating how they were calculated

Our web collection on statistics for biologists contains articles on many of the points above.

Software and code

Policy information about availability of computer code

Data collection	No software was used to collect data in this study.
Data analysis	Analyses were performed with Python v3.10.5 and R v.2.2. Key python packages include: abagen==0.1.3, brainspace==0.1.10, neuromaps==0.0.3. Full details of all packages, a Dockerfile and link to docker image, and all code used for these analyses are publicly available at https://github.com/richardajdear/AHBA_gradients.

For manuscripts utilizing custom algorithms or software that are central to the research but not yet described in published literature, software must be made available to editors and reviewers. We strongly encourage code deposition in a community repository (e.g. GitHub). See the Nature Portfolio guidelines for submitting code & software for further information.

Data

Policy information about availability of data

All manuscripts must include a data availability statement. This statement should provide the following information, where applicable:

- Accession codes, unique identifiers, or web links for publicly available datasets
- A description of any restrictions on data availability
- For clinical datasets or third party data, please ensure that the statement adheres to our policy

Gene expression datasets used are all publicly available:
• The Allen Human Brain Atlas is available at http://human.brain-map.org, and individual donor HCP-MMP parcellation images at https://figshare.com/articles/

dataset/AHBAdata/6852911.
• The BrainSpan Atlas is available at https://www.brainspan.org/.
• The Allen Human Cell Atlas is available at https://portal.brain-map.org/atlases-and-data/rnaseq.
• The PsychENCODE dataset is available at https://github.com/dhglab/Broad-transcriptomic-dysregulation-across-the-cerebral-cortex-in-ASD.

Neuroimaging maps of healthy brain features are available in the neuromaps package (https://github.com/netneurolab/neuromaps). For convenience all brain maps used are provided in Supplementary Table 3-4. Gene lists used for enrichment analyses were all obtained from prior publications as detailed in Methods.

Human research participants

Policy information about studies involving human research participants and Sex and Gender in Research.

Reporting on sex and gender	Sex-and gender-based analysis was not performed due to lack of sufficient sample size in the AHBA dataset (n=6, 1 female).
Population characteristics	The AHBA dataset brains have mean age of 42.5 (SD 13.4).
Recruitment	Recruitment of the AHBA data was as detailed on the Allen Institute website. Specifically, postmortem tissue from males and females between 18 – 68 years of age and no known history of neuropsychiatric or neurological conditions ('control' cases) were eligible for inclusion.
Ethics oversight	Ethics oversight was performed by the Allen Institute for Brain Science.

Note that full information on the approval of the study protocol must also be provided in the manuscript.

Field-specific reporting

Please select the one below that is the best fit for your research. If you are not sure, read the appropriate sections before making your selection.

☒ Life sciences ☐ Behavioural & social sciences ☐ Ecological, evolutionary & environmental sciences

For a reference copy of the document with all sections, see nature.com/documents/nr-reporting-summary-flat.pdf

Life sciences study design

All studies must disclose on these points even when the disclosure is negative.

Sample size	No sample size calculation was performed as the AHBA dataset comprises only 6 brains; instead, our analysis proposed analysis by disjoint triplets as a proxy for generalisability. To ensure that this sample size was sufficient for the analysis performed, we reproduced and validated the results in three other transcriptomic datasets representing the three main dimensions of variability: developmental variability (BrainSpan), cell-type variability (Allen Cell Atlas), and inter-indidvidual variability (PsychENCODE).
Data exclusions	In the BrainSpan dataset, the 7 donor brains from which fewer than 4 regions were sampled were excluded from analysis due to insufficient spatial representation to normalise by donor, leaving 35 donor brains. In the PsychENCODE dataset, samples with RIN < 3 were excluded from analysis.
Replication	While replication was not possible as the AHBA is the only transcriptomic data of the entire human cortex with high spatial resolution, we performed one replication of the three components in external RNAseq data from the PsychENCODE consortium.
Randomization	Randomization was not relevant to our analysis as we were identifying generalisable transcriptomic patterns across all human brains, for which we proposed the disjoint triplets analysis as a proxy for generalisability.
Blinding	Blinding was not relevant to our study as we did not perform randomized experiments.

Reporting for specific materials, systems and methods

We require information from authors about some types of materials, experimental systems and methods used in many studies. Here, indicate whether each material, system or method listed is relevant to your study. If you are not sure if a list item applies to your research, read the appropriate section before selecting a response.

Materials & experimental systems

n/a	Involved in the study
☒	☐ Antibodies
☒	☐ Eukaryotic cell lines
☒	☐ Palaeontology and archaeology
☒	☐ Animals and other organisms
☒	☐ Clinical data
☒	☐ Dual use research of concern

Methods

n/a	Involved in the study
☒	☐ ChIP-seq
☒	☐ Flow cytometry
☒	☐ MRI-based neuroimaging
